# Walnuts, Long-Chain Polyunsaturated Fatty Acids, and Adolescent Brain Development: Protocol for the Walnuts Smart Snack Dietary Intervention Trial

**DOI:** 10.3389/fped.2021.593847

**Published:** 2021-06-08

**Authors:** Jordi Julvez, Florence Gignac, Silvia Fernández-Barrés, Dora Romaguera, Aleix Sala-Vila, Otavio T. Ranzani, Cecilia Persavento, Anna Delgado, Albert Carol, Jaume Torrent, Judith Gonzalez, Eduard Roso, Jose Barrera-Gómez, Mónica López-Vicente, Raquel Garcia-Esteban, Olivier Boucher, Joan Forns, Miguel Burgaleta, Nuria Sebastián, Josefina Canals, Victoria Arija, Xavier Basagaña, Emilio Ros, Joan Vendrell, Jordi Salas-Salvadó, Jordi Sunyer

**Affiliations:** ^1^Institut d'Investigació Sanitària Pere Virgili, Hospital Universitari Sant Joan de Reus, Reus, Spain; ^2^ISGlobal- Instituto de Salud Global de Barcelona-Campus MAR, Parc de Recerca Biomèdica de Barcelona (PRBB), Barcelona, Spain; ^3^CIBER Epidemiología y Salud Pública, Instituto de Salud Carlos III, Madrid, Spain; ^4^Universitat Pompeu Fabra, Barcelona, Spain; ^5^Instituto de Investigación Sanitaria Illes Balears, Hospital Universitari Son Espases, Palma, Spain; ^6^CIBER Fisiopatología de la Obesidad y Nutrición, Instituto de Salud Carlos III (ISCIII), Madrid, Spain; ^7^Barcelonaßeta Brain Research Center, Pasqual Maragall Foundation, Barcelona, Spain; ^8^IMIM-Hospital del Mar Medical Research Institute, Barcelona, Spain; ^9^Centre de Recherche du Centre Hospitalier de l'Université de Montreal, Montreal, QC, Canada; ^10^Nutrition and Public Health Unit, Research Group on Nutrition and Mental Health, (NUTRISAM), Faculty of Medicine and Health Science, Universitat Rovira i Virgili, Reus, Spain; ^11^Lipid Clinic, Endocrinology and Nutrition Service, Hospital Clínic, Biomedical Research Institute August Pi i Sunyer (IDIBAPS), Barcelona, Spain; ^12^Centro de Investigación Biomédica en Red de Diabetes y Enfermedades Metabólicas Asociadas, Instituto de Salud Carlos III, Madrid, Spain; ^13^Human Nutrition Unit, Universitat Rovira i Virgili, Department of Biochemistry and Biotechnology, Reus, Spain

**Keywords:** neuropsychological development, dietary intervention, walnuts, omega-3 PUFA, ALA, randomized controlled trial, adolescence

## Abstract

**Background:** Adolescence, when the most complex behaviors are refined to adult sophistication, represents a major window of opportunity and vulnerability for neuropsychological development. To support and protect this complex and active brain growth, different nutritional components considered essential need to be acquired from the diet. For instance, omega-3 fatty acids are mainly obtained from seafood, seeds, and walnuts. Known for their rich lipid profile, walnuts contain sizable amounts of an essential fatty acid, alpha-linolenic acid (ALA), the vegetable omega-3 fatty acid that is the precursor of two longer-chain omega-3 polyunsaturated fatty acids (omega-3 PUFA): docosahexaenoic (DHA) and eicosapentaenoic (EPA) acids. While there is growing evidence of neuropsychological improvements in the young developing brain associated with omega-3 PUFA intake, few studies have examined whether consuming walnuts during adolescence entails similar beneficial effects. There is a need to further explore the ways in which walnuts influence youthful brain function, particularly for the long-term. Thus, we designed the WALNUTs study (WSS), a population-based randomized controlled trial conducted in adolescents in Barcelona, Spain. We hypothesize that walnut intake will increase omega-3 PUFA tissue availability (particularly ALA) to a level that enhances the neuropsychological development during adolescence.

**Methodology/Design:** We conducted a 6-month population-based randomized controlled trial in teenagers (*n* = 800) and we aimed to determine the effectiveness of the intervention (four walnuts per day, or 30 kernel g, ~1.5g of ALA) in enhancing brain neuropsychological and socio-emotional development compared to a control group with no walnut intervention. Before randomization, different neuropsychological tests were recorded for all participants, and blood samples (in a subsample of participants) were collected to measure omega-3 PUFA levels at baseline, and all again, after randomization and the intervention. The data is now collected and we will conduct linear regression models to assess the effect of the intervention.

**Discussion:** The WALNUTs (WSS) study results will allow us to better understand the role of plant-based omega-3 PUFA intake from regular walnut consumption on neuropsychological development during adolescence. Results could be translated into nutritional public health recommendations targeting teenagers.

**Trial Registration:** ClinicalTrials.gov, U.S. National Library of Medicine, National Institutes of Health # NCT02590848. Retrospectively registered 29/10/2015.

## Background

Adolescence is a critical period for brain development, mainly because the prefrontal cortex, which is responsible for important functions such as logical thinking, working memory, and organizing skills, is the last region of the brain to mature around the early twenties (1). Adolescence is also a time of refinement of brain connectivity and complex behaviors. Indeed, it is a critical period for the development of psychological and psychiatric pathologies, such as substance abuse, schizophrenia, and mood and anxiety disorders (1–3).

The brain is a highly active organ and its development and metabolism require a large amount of energy and nutrients (4, 5). In this context, lack of essential nutrients may interfere with brain development in youth. Therefore, enhancing these complex processes, such as by adopting a healthy and balanced diet providing essential nutrients, may have long-term functional consequences (6).

Making up ~15 to 30% of the brain's dry weight (7), PUFAs are an example of essential and semi-essential nutritional components that are mostly obtained through the diet (mainly from seafood, seeds, and walnuts) (8). Three of these PUFAs play an essential role in brain development: long-chain omega-3 acids—docosahexaenoic acid (DHA) and eicosapentaenoic acid (EPA)—and the omega-6 acid arachidonic acid (AA). Experimental and clinical studies have demonstrated the importance of PUFAs in the function and architecture of the central nervous system throughout various stages of life, from neural development to neurodegeneration (4, 7). In the adolescent population, several large randomized controlled trials of supplementation with DHA and EPA have reported a favorable effect on neuropsychological and behavioral outcomes (9, 10). Thus, a large randomized controlled trial conducted in healthy young adults using computerized cognitive tests found that reaction time latencies and working memory were improved after 6 months of DHA supplementation (11).

Of all edible plants, walnuts are among the richest in the vegetable omega-3 fatty acid and alpha-linolenic acid (ALA), the precursor for DHA and EPA (12, 13). However, ALA is poorly transformed to EPA, while ALA itself has shown positive effects on brain function (14). Walnuts are also rich in fiber, vitamins, minerals, and other bioactive compounds capable of improving brain health (12). Short-term walnut consumption has been able to increase peripheral levels of EPA in humans (15), and different experimental studies have pointed to potential benefits in terms of cognition. Studies of walnut-fed rats fed showed improvements in working memory (12), and in humans, a parallel-group randomized controlled trial carried out among 447 older adults (mean age, 66.9 years) from Spain, found that a Mediterranean diet supplemented with 30g/day mixed nuts (including 15 g of raw, unprocessed walnuts) improved memory and delayed age-related cognitive decline (16).

Nut studies examining cognitive outcomes in young people are scarce. An observational cohort study assessed the association of various foods on the cognitive function of children and adolescents and reported a beneficial effect of nut consumption on visual attention and processing (17). One small double-blind, randomized, placebo-controlled crossover (8-week intervention and 6-week washout) trial with walnuts carried out with young college students aged 18 to 25 years showed improvements in inferential verbal reasoning (18). However, no experimental study focused on the effects of walnut consumption on adolescents' cognitive functions. Furthermore, a recent large observational study with NHANES data of 26,656 adult participants also found a protective association of walnut intake against depressive symptom scores (19).

In view of these preliminary findings, there is a need to further explore the potential brain development benefits of walnut consumption during adolescence, a period when the most complex behaviors are develop into adult sophistication. Moreover, adolescents represent a population that tends to be less targeted by health studies investigating the effects of environmental exposure on brain development (6). By conducting a population-based randomized controlled trial, the WALNUTs (WSS) study aims to assess whether walnut consumption has potential beneficial effects on teenage brain function using different neuropsychological and behavioral assessments.

We hypothesized that walnut supplementation for 6 months would enhance neuropsychological and behavioral (socio-emotional) development among healthy teenagers. We further hypothesized that a walnut intervention for 6 months would increase omega-3 PUFA tissue availability.

## Methods and Materials

### Trial Design

**Figure 1:**
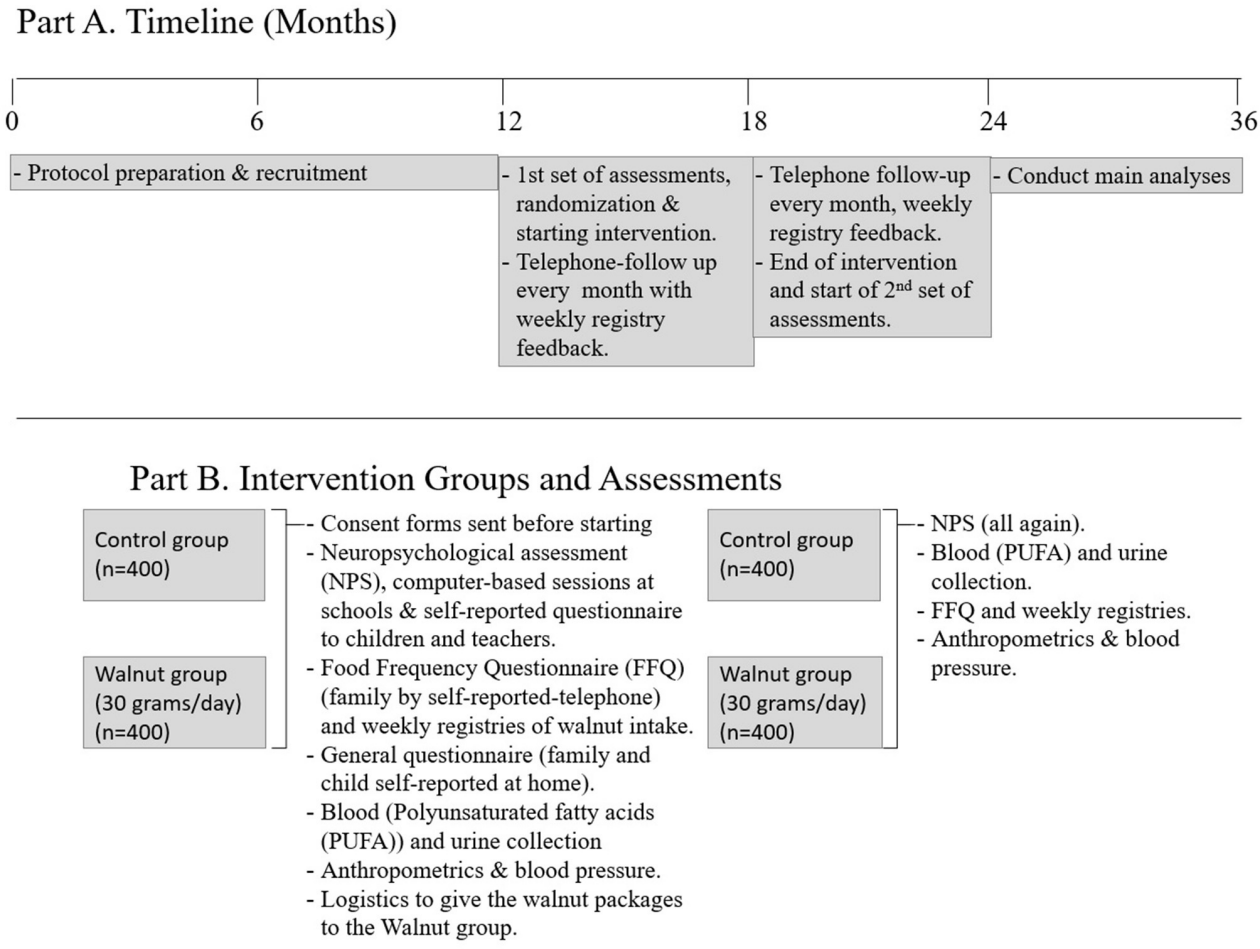
Project design and structure.

WALNUTs (WSS) is a multi-school, parallel (two-group), controlled, 6-month superiority randomized clinical trial in a large population-based sample aiming to include 800 healthy teenagers in Barcelona, Spain. After data collection, 771 participants were equally randomized in two groups (386/385), but because of some exclusions post-randomization, 748 were finally included. Study subjects were randomly assigned to two intervention groups just after baseline assessments: the walnut group (*n* = 370, consuming walnuts daily and advised to follow general healthful eating recommendations, such as eating a piece of fresh fruit every day) or control group (*n* = 378, not consuming walnuts and advised also to follow the same general healthful eating recommendations). They were assessed at baseline and after the intervention by several internationally validated neuropsychological tests and behavioral rating scales. Due to the project's budget limitations, a random subsample of 200 adolescents per group of intervention (*n* = 400) were targeted to measure changes in omega-3 PUFA biomarkers. We finally got 270 participants, 139 in the walnut group and 131 in the control group. All participants randomized to the walnut group received 30g/day of kernel walnuts to be incorporated into their diet for free. The type of walnuts selected, Californian walnuts, is estimated to contain about 9 g of ALA per 100 g (20, 21). There is evidence that young Spanish subjects between 10–13 and 14–17 years of age consume on average 5.7 g (SD 24.5 g) and 6.1 g (SD, 20.8 g) of nuts daily (22), which supports the feasibility of our trial design. Figure 1 shows the project's design and structure.

### Eligibility Criteria

The participants eligible were adolescents aged between 11 and 16 years attending regular schools in Barcelona. Originally, the study protocol was designed to recruit adolescents aged 12–15 years; however, the schools facilitated participant recruitment through the classes, and there were children a bit younger and older that we did not want to exclude if they were willing to participate in the study. Exclusion criteria include those consuming supplements of omega-3 PUFAs regularly, eating walnuts on a daily basis, and/or having an allergy to walnuts and/or gluten. Subjects were excluded also if they reported lactose intolerance or an allergy to cereals, dried fruits, peanuts, soy, sesame, or sulfites, since the walnut industry manipulates these products and there could be traces of them in the walnut packages. Eligible candidates who were siblings of family units were randomized and allocated to the same group and analyzed as clusters.

### Sample Size

Prior to data collection, the statistical power of the study for neuropsychological outcomes was based on 400 participants per group. Six primary outcomes were considered: the N-back task, the Attention Network Test (ANT), the Tests of Primary Mental Abilities (PMA-R, the Spanish adaptation), the Roulettes Task (adapted from the Cups Task), the Strengths and Difficulties Questionnaire (SDQ), and the Attention Deficit Hyperactivity Disorder (ADHD) DSM-IV form list. The cognitive outcomes with mean values of 100 (SD 15) units corresponding to the standard values of neuropsychological scores in the general population (23) were considered, with a correlation between them of 0.25. The targeted intervention effect was a change of 3 units, based on previous studies in adult samples (16, 18). We considered a type I error of 0.05 and corrected calculations for multiplicity using the Benjamini-Hochberg method. A 10% loss of follow-up was assumed. Additionally, we assumed that the final models had an R^2^ of 20%. With all these considerations, the study would have 95% power to detect the effect with at least one outcome, 90% to detect the effect with at least two, 80% to detect the effect with at least 3, 70% to detect the effect with at least 4, 55% to detect the effect with at least 5, and 31% to detect the effect with all six outcomes. Similar power calculations can be made from the final participation sample of 748 participants.

### Procedures

#### Recruitment and Randomization

An experienced fieldwork technician with the help of two more experts recruited participants in collaboration with the Barcelona school system. We first obtained permission from the boards of several schools in Barcelona to inform them about the trial. Then, we provided the schools with a recruitment fllier to assess willingness to collaborate and grant us permission to contact interested families by telephone. Families interested in participating contacted us through the school and filled out a form with their telephone number. Trained personnel contacted the parents and explained the study in detail. Fieldworkers verified the eligibility criteria of each candidate. We performed age-, gender-, and maternal education-stratified random computerized sampling within each school to assign adolescents to one of the two groups. In order to avoid contamination, we gave the walnuts for free to the corresponding group and asked participants to consume them at home.

#### Implementation and Adherence

All families received a basic guide to the seasonal fruits needed to follow healthful diet recommendations (eating a piece of fresh fruit every day) in order to ensure implementation and adherence. The control group received the same instructions, including the general recommendation to eat a piece of fresh fruit every day, as well as feedback to encourage them to remain in the study. Families in the walnut intervention group received additional instructions on how to encourage participants to eat their daily allotment of walnuts, which we supplied for free in sachets of 30 grams; no other recommendations were given. We asked parents to supervise the intervention by checking the subject's adherence to the walnut consumption. To evaluate adherence to the intervention, the Survey Monkey web-based platform was used weekly. Participants in the walnut group were contacted in the middle of the study, and recipes for dishes containing walnuts were provided; they were also asked to report their daily walnut consumption weekly, using the Survey Monkey platform and to cross off the days they had or hadn't eaten the allocated amounts of walnuts on a calendar, in order to evaluate adherence to the intervention. Moreover, the participants were eligible for a periodic raffle for completing the Survey Monkey reports weekly. The prize was entrance tickets to a science museum.

## Results

### Dietary Assessment

A food frequency questionnaire (FFQ), validated for the Spanish population (24) and adapted to the adolescent range, was administered to all adolescent participants at baseline and at the end of the intervention (after 6 months). Additionally, a short FFQ was completed every 3 months. We added the measurement of dietary patterns to our study in order to control for these factors in secondary analyses, since diet can be changed after a nutritional intervention.

### Neuropsychological and Behavioral Testing

Multiple primary endpoints concerning the neuropsychological and behavioral development of adolescents was assessed before randomization, at baseline (pre-intervention) and after 6 months (post-intervention). A total of six tests (six primary outcomes) developed for assessing children and adolescents were used to measure changes from baseline in the main score for each of the tests for neuropsychological (working memory, attention, fluid intelligence and executive function) and behavioral (socio-emotional and ADHD symptoms) outcomes (25). The first primary outcome measurement was the N-back task, which is a computer-based test assessing working memory using series of numbers. The stimulus appears in the laptop screen one by one. Children were invited to press a button in the keyboard when the stimulus is the same as the previous one in the 1-back level. In 2-back, they were required to press the button when the stimulus in the screen is the same as the one presented two trials back. In 3-back, they had to press when the stimulus was the same as the one presented three trials back. The outcome is *d*: *d* prime (*d'*) = *z* (hit rate) - *z* (false alarm rate) (26). The second measurement was the Attention Network Test (ANT), also known as the flanker test, which is also a computerized test aimed at examining sustained attention based on reaction times to target stimuli. The test provides several attention functions (orienting, alerting, and conflicting); however, hit reaction time standard error is the “gold standard” to measure sustained attention, since it is the reaction time variability during the attention task (27, 28). The third measurement was the inductive reasoning subtest (fluid intelligence) of the Tests of Primary Mental Abilities (PMA-R, the Spanish adaptation) (29). The fourth measurement, the Roulettes Task (adapted from the Cups Task), was a gambling task aiming to assess risky decision making, i.e., whether the participants adjust their risky behavior according to the probabilities and importance of the outcome (cognition influenced by emotion) (30). The fifth measurement was the self-reported version of the Strengths and Difficulties Questionnaire (SDQ). It consists of 25 questions, organized in a general score of problem behavior and five subscales aimed to assess emotional symptoms, conduct problems, hyperactivity/inattention, peer relationship problems and pro-social behavior (31). The sixth measurement was the Attention Deficit Hyperactivity Disorder (ADHD) DSM-IV form list. We asked the teacher of the adolescent to answer 18 multiple-choice questions in order to rate his/her ADHD performance. The scores were transformed into a number of symptoms and diagnostic criteria for ADHD and inattentive and hyperactive subtypes. The administration of these neuropsychological tests was carried out by a trained psychologist and fieldwork technicians, and the tests were hold at the schools after securing comfortable and quiet quarters.

### Laboratory Determinations

The venipuncture blood collection was done by a nurse at the schools after securing appropriate conditions at baseline and after 6 months of intervention. The red blood cell proportions of omega-3 fatty acids (DHA, EPA and ALA) and omega-6 fatty acids (AA), among other fatty acids, were determined by gas chromatography at baseline and after 6 months of intervention. Details on quality control for this method are described elsewhere (32). The results were expressed in relative amounts (percent of total fatty acids).

### Clinical Evaluation and Other Secondary Measurements

In addition to the neuropsychological and behavioral endpoints, secondary intermediate outcomes of interest were measured at baseline and after 6 months of intervention. We aim to explore whether there are changes in height (in cm), weight (in kg), waist circumference (in cm), and blood pressure. These other health outcomes can be affected by changes in the diet: particularly walnut intake can improve cardiometabolic indicators (33). Height was measured using the stadiometer model SECA 214, weight by the weighing scales SECA 770 model, and waist circumference with the SECA 201 tape model. Blood pressure (systolic and diastolic) were measured (in triplicate) after 5 min of rest with an OMROM 705-CPII device in the dominant arm. All measurements were done by a trained nurse at the schools and followed standard procedures.

Additionally, at baseline, two questionnaires of sociodemographic and clinical characteristics and lifestyle factors were obtained to collect covariables such as parental education and socio-economic level, parental mental health status, sociodemographic characteristics, adolescent health history, and lifestyle habits such as physical activity, sleep duration, and daily screen time exposure. Further estimations of environment and ambient pollution based on home and school geo-localizations were recorded.

Finally, in the same subsample of 270 participants, we collected extra samples of blood and urine before and after the nutritional intervention to assess potential secondary effects of the intervention and also to investigate the biological effects of other important risk factors collected in this study, such as lifestyle factors, environmental exposure, nutritional status, and the mental health status of the participants.

## Data Analysis

Descriptive statistics (frequencies, means, standard deviations for normally distributed data; medians and interquartile ranges for nonparametric data) will be used to describe the baseline characteristics of the walnut and control groups. To assess the effect of the intervention on brain development, we use multivariable linear regression models. Similar regression models will be used to assess the effect of the intervention on red blood cell PUFA proportions, particularly ALA, as outcome variables. To assess the proportion of the effect of the intervention on brain development that is mediated by red blood cell PUFA changes, results will be compared in models with and without the mediator. All analyses will be based on the intention to treat in order to estimate the effectiveness of a public health recommendation (i.e., to encourage eating 30 g of walnuts per day). We also plan to conduct a per-protocol analysis with those who comply with the intervention, i.e., single days of reporting eating walnuts (≥ 100 days during 6 months) (34). The nominal statistical significance will be set at the *P* < 0.05 level (two-sided) for the primary outcomes. Statistical analyses are performed with STATA. A detailed statistical analysis plan will be established before database lock and any analyses.

Using multiple primary endpoints, the study will be considered successful if the results of either one of the primary endpoints is statistically significant in favor of the experimental treatment and/or treatment adherence. In this case, we will report for each primary endpoint a nominally significant level and the corrected *p*-value determined by the method for multiplicity adjustment.

## Discussion

To the best of our knowledge, this is the first medium-term randomized intervention trial aiming to assess the effect of walnut consumption on adolescent brain development. The selected battery of neuropsychological and behavioral tests will provide the opportunity to enrich the interpretation of the findings. However, the design of this study is not exempt from potential limitations. First, the study selected walnuts for their richness in essential fatty acids, particularly ALA, with the hypothesis that they have an effect on the young developing brain. However, using walnuts will make the true PUFA content of the diet less consistent than if administered via a daily pill supplement. While the PUFA concentrations of California walnuts are fairly stable, we will minimize this potential bias by standardizing walnut dosage and type for the duration of the intervention. In any case, we measured the ALA biomarker in blood before and after the intervention as objective proof of adherence to the intervention. Second, performing a double-blind dietary trial, the gold standard of clinical trials, is problematic and known to be an important limitation in feeding studies of whole foods (35, 36). However, investigators performing biochemical determinations and statistical analyses are blinded to the intervention. Third, ensuring compliance of adolescents with daily walnut consumption for a period of 6 months is difficult and may result in lack of adherence and losses to follow-up. To offset this limitation, we planned to contact participants in the middle of the study, distribute recipes for dishes containing walnuts, ship walnuts to subjects' homes or schools for free, and ask the parent/tutor to supervise the intervention. In summary, this randomized feeding trial intends to focus on nutritional and neuropsychological sciences in the adolescents, a population group infrequently evaluated in clinical and public health research (https://www.nature.com/collections/vbmfnrsssw).

## Data Availability Statement

The original contributions presented in the study are included in the article/supplementary material, further inquiries can be directed to the corresponding authors.

## Ethics Statement

The studies involving human participants were reviewed and approved by CEIC Parc Salut Mar (approval number: 2015/6026/I). Written informed consent to participate in this study was provided by the participants' legal guardian/next of kin.

## Author Contributions

JJ, SF-B, and DR developed the study protocol and/or sections of the study protocol. JJ, SF-B, and DR revised the study protocol and/or sections of the study protocol. JJ, FG, SF-B, DR, and OR drafted the manuscript. CP, AD, AC, JT, JG, and ER were involved in the fieldwork of the study. All authors revised the manuscript for important intellectual content, approved the version to be published, and agreed to be accountable for all aspects of the work.

## Conflict of Interest

JJ, AS-V, and EmR have received grants for research through their institutions from the California Walnut Commission (CWC), Folsom, California. ER has also received honoraria for consulting and presentations from the CWC and is a nonpaid member of its Scientific Advisory Council, besides having received honoraria for presentations from Danone JS-S reports serving on the board of the International Nut and Dried Fruit Council (nonpaid member of the scientific committee) and receiving grant support from this entity through his institution. He also reports serving on the Executive Committee of the Instituto Danone Spain. He has also received research funding (walnuts, olive oil, hazelnuts, and almonds for the PREDIMED study) from the CWC; Patrimonio Comunal Olivarero, Spain; La Morella Nuts, Spain; and Borges S.A., Spain, respectively. He reports receiving consulting fees or travel expenses from Nuts for Life and the Australian Nut Industry Council. All remaining authors declare that the research was conducted in the absence of any commercial or financial relationships that could be construed as a potential conflic of interest.

## Abbreviations

AA: arachidonic acid
ADHD: attention deficit hyperactivity disorder
ANT: attention network test
PUFAs: long chain polyunsaturated fatty acids
ALA: alpha-linolenic acid
EPA: eicosapentaenoic acid
DHA: docosahexaenoic acid
SDQ: strengths and difficulties questionnaire
WSS: walnuts smart snack study.
